# Regulation of autophagy fires up the cold tumor microenvironment to improve cancer immunotherapy

**DOI:** 10.3389/fimmu.2022.1018903

**Published:** 2022-10-10

**Authors:** Zhicheng Jin, Xuefeng Sun, Yaoyao Wang, Chao Zhou, Haihua Yang, Suna Zhou

**Affiliations:** ^1^ Key Laboratory of Radiation Oncology of Taizhou, Radiation Oncology Institute of Enze Medical Health Academy, Department of Radiation Oncology, Taizhou Hospital Affiliated to Wenzhou Medical University, Zhejiang, China; ^2^ Fuwai Hospital, Chinese Academy of Medical Sciences & Peking Union Medical College/National Center for Cardiovascular Diseases, Beijing, China; ^3^ Department of Radiation Oncology, Xi’an No.3 Hospital, the Affiliated Hospital of Northwest University, Xi’an, China

**Keywords:** autophagy, tumor microenvironment, immune cells, immunogenic cell death, antigen presentation, immunotherapy

## Abstract

Immunotherapies, such as immune checkpoint inhibitors (ICIs) and chimeric antigen receptor (CAR) T cells, have revolutionized the treatment of patients with advanced and metastatic tumors resistant to traditional therapies. However, the immunosuppressed tumor microenvironment (TME) results in a weak response to immunotherapy. Therefore, to realize the full potential of immunotherapy and obstacle barriers, it is essential to explore how to convert cold TME to hot TME. Autophagy is a crucial cellular process that preserves cellular stability in the cellular components of the TME, contributing to the characterization of the immunosuppressive TME. Targeted autophagy ignites immunosuppressive TME by influencing antigen release, antigen presentation, antigen recognition, and immune cell trafficking, thereby enhancing the effectiveness of cancer immunotherapy and overcoming resistance to immunotherapy. In this review, we summarize the characteristics and components of TME, explore the mechanisms and functions of autophagy in the characterization and regulation of TME, and discuss autophagy-based therapies as adjuvant enhancers of immunotherapy to improve the effectiveness of immunotherapy.

## 1 Introduction

Current prognoses for individuals with advanced cancer are generally poor, making cancer the second most common cause of global deaths (1). Immunotherapy has recently improved survival advantages over traditional treatments for various tumor types, especially advanced non-small cell lung cancer (NSCLC) and melanoma. However, how to raise response rates is still an urgent issue (2). Although immunotherapy combined with chemotherapy, targeted medication, or radiotherapy has been confirmed to finitely improve the anti-tumor effect, it is more necessary to explore other safe and efficient immunosensitizers or combination regimens (3, 4).

**Figure 1:**
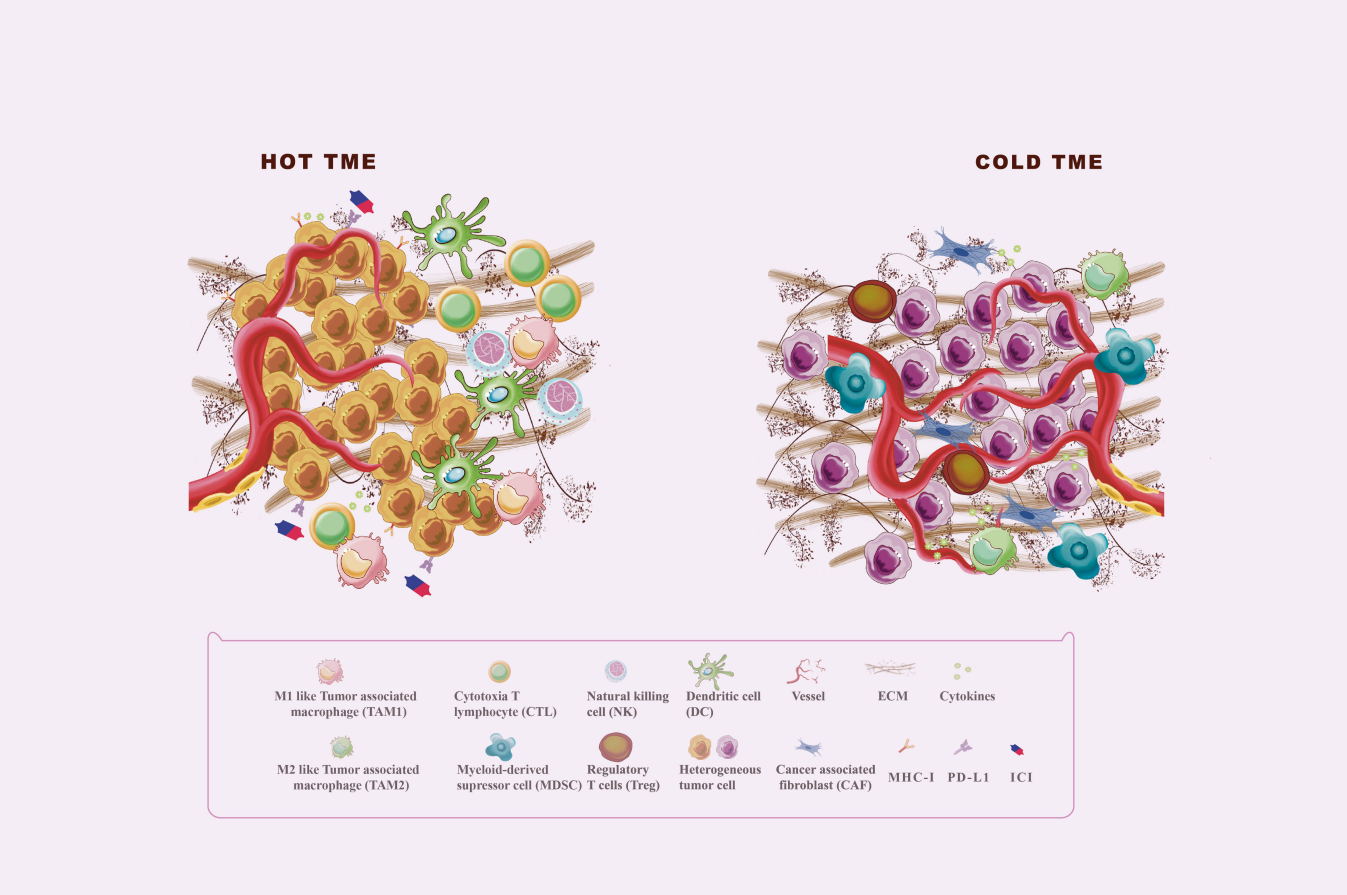
Hot and cold TME. The tumor microenvironment (TME) is divided into the immune desert (cold) and immunoinflammatory (hot) phenotypes. In the immune desert phenotype, the absence of T cells in the tumor may be due to the lack of tumor antigens and antigen-presenting cells (APC), the secretion of immunosuppressive molecules such as TGF-β and IL-10, and the infiltration of immunosuppressive cells including MDSC, M2 macrophages, and Treg. In addition, abnormal angiogenesis and excessive ECM also play an important role in the formation of cold TME. The immunoinflammatory phenotype is thought to be a prominent infiltration of cytotoxic T lymphocytes (CTLs) in the core of the TME, with activated antigen-presenting cell (APC) markers and type 1 interferon (IFN) responses.

Tumor immunogenicity deficiency and immunosuppressive tumor microenvironment (TME) formation are the major causes of immunotherapy ineffectiveness or resistance (5). TME is a complex ecosystem made up of extracellular soluble compounds, stromal cells, immune cells, aberrant vascular networks, tumor cells, and dynamic oxygen content (6). Died tumor cells release antigens, triggering traditional antigen-presentation dendritic cells (DC) to catch and present major histocompatibility complex (MHC) class I-antigens to T cells, followed by immune activation mediated by the activated CD8^+^T cells (7). Effector CD8^+^ T cells, also named cytotoxic T lymphocytes (CTL), play the most significant adaptive tumor-killing effects in TME by releasing cytotoxic perforins, granzymes, and cytokines such as interferon (IFN)-γ and tumor necrosis factor-α (8). Natural killer (NK) cells act as major MHC I-independent tumor-killing immune cells and are the important complement of T cell-mediated antitumor immunity (9). However, the anti-tumor immunity will be weakened by immunosuppressive TME formed by recruiting immunosuppressive cells, such as Myeloid-derived suppressor cells (MDSC) and regulatory T cells (Treg) (10, 11). Tumor-associated macrophages (TAMs), accounting for 50% of infiltrating tumor stromal cells, are major immune cells in TME with phenotypically heterogeneous and functionally diverse. M1 macrophages can secrete proinflammatory cytokines, increase tumor antigen presentation as well as directly kill tumor cells through phagocytosis, resulting in immune activation (12). Moreover, M2 macrophages exert pro-tumor function by producing cytokines, such as interleukin (IL)-10 and transforming growth factor (TGF)-β, and play an immunosuppressive effect by the expression of programmed cell death ligand (PD-L1) and PD-L2 directly inhibiting cytotoxic T cell functions (12). As shown in **Figure 1**, TME could be shifted between “Hot” and “Cold” TME by recruiting immunosuppressive or immunostimulatory cells, which affect the response of tumors to immunotherapy (13). “Hot” TME is characterized by a prominent infiltration of CTLs, high expression of PD-L1 on tumor cells, upregulation of antigen-presenting cells (APC) markers, and activation of type 1IFN responses that all help to enhance the response to immunotherapy (13, 14). “Cold” TME is also known as an “immune desert” or “immune rejection” TME, and is characterized by rare CTLs infiltration, extensive fibrosis, abnormal vessel structure, and redundant immunosuppressive cells infiltrated, as well as low MHC I expression (14). The main mechanisms of TME regulation include antigen release, presentation, recognition of antigens, and recruitment and outflow of immune cells. Reprogramming the immunosuppressive TME into the immunostimulatory phenotype may enhance the response sensitivity of tumors to immunotherapy (6, 14, 15).

Autophagy is a process by which either unfolded/misfolded/damaged protein aggregates or organelles are eradicated for the maintenance of cellular homeostasis (16). US Food and Drug Administration (FDA)-approved autophagy inhibitors hydroxychloroquine (HCQ) and chloroquine (CQ) have recently shown clinical benefits in the treatment of cancer patients when combined with chemotherapy, radiotherapy, or monotherapy (17). Altered autophagy in cancer, immune or stromal cells can regulate tumor-immune interactions to remodel the TME (18). Some studies have manifested that combination treatments of autophagy-targeted medicines and immunotherapy boost anti-tumor immune efficacy and enhance clinical benefits (18). Although autophagy-targeted medicines combined with immunotherapy are potential treatments, the precise mechanisms of which are still in the exploratory phase.

This study provides a comprehensive and in-depth review of the role of autophagy in TME and tumor immunotherapy. It gives insights into the role of autophagy in TME regulation, suggesting that immunotherapy will be improved through the exploration of either autophagy-based immunosensitizers or combination therapies.

## 2 TME remodeling is a critical influencer in immunotherapy

Immunotherapies exhibit CTLs-dependent tumor-killing effects and are popularly applied in clinical treatment. TME without infiltration of T cells or with T cells exhaustion is the biggest obstacle to response to immunotherapy for cancer patients. Hypoxia, abnormal vasculature, and alteration in the three-dimensional stromal environment are the three most important characteristics of TME. These characteristics mainly impair the priming and infiltration of T cells, resulting in the exhaustion of T cells forming an immunosuppressive TME.

### 2.1 Current cancer immunotherapy

Currently, cancer immunotherapies fully utilize the immune system to eradicate tumor cells. Two notable and successful immunotherapies are immune checkpoint inhibitors (ICIs) and chimeric antigen receptor (CAR) T cells. Due to factors such as the extracellular matrix (ECM), current CAR-T cells are only effective against hematological tumors, toxic at high doses, and cannot simultaneously target multiple antigens (19). The failure of T cells to penetrate the TME and their concomitant exhaustion significantly impact ICI therapies (20). Either the innate or acquired immunosuppressive TME seriously impairs the efficacies of immunotherapies (21).

### 2.2 Characteristics of TME

The TME comprises tumor cells, immune cells, stromal cells, vascular endothelial cells (ECs), and their non-cellular components such as the extracellular soluble molecules and ECM, along with vascular networks (6). These complex networks of cells and non-cellular components regulate the functions of immune cells within the tumor, consequently impacting the efficacy of immunotherapies (6). Furthermore, the TME is a dynamic network structure that changes with either cancer progression or the administration of various treatments (6). Hypoxia-caused three-dimensional stromal environment alteration and aberrant vascular construction affect the communication among tumor, immune and stromal cells, dynamically altering the TME into a cold state.

#### 2.2.1 Hypoxia

As the tumor grows, the pre-existing vasculature cannot satisfy its perfusion, leading to declining oxygen levels and the formation of a hypoxic environment (22). Hypoxia-inducible factor-1 (HIF-1), produced by either tumor or TME-associated cells, is a significant regulator of hypoxia. It then stimulates the expression of numerous genes involved in establishing immunosuppressive TME (22). Activated HIF-1 signaling attenuates MHC-I antigen presentation and hampers the infiltration and cytotoxic functions of the effector T cells and NK cells (22, 23). And, enhanced HIF-1 promotes the development and recruitment of immunosuppressive cells including M2-type macrophages, MDSC, and Treg (22, 23). Furthermore, HIF-1-mediated elevated expression of PD-L1 and CD47 contributes to the exhaustion of CTL and inactivation of phagocytosis (22, 23). Negative regulatory genes of the anti-tumor immune were found to be largely elevated in hypoxia TME, associated with the malignant phenotype of the tumors and poor patient prognosis (24). Interestingly, HIF-1 signaling stimulates angiogenesis by boosting pro-angiogenic factors, influencing another significant characteristic of TME (25).

#### 2.2.2 Abnormal vasculature

The harsh TME often results in disrupted blood flow and oxygen-/nutrient-poor blood perfusion. Hypoxia demarcates the start of angiogenesis. HIF-1 is an upstream regulatory molecule of vascular endothelial growth factor (VEGF), by which and its receptor on Ecs, VEGFR2, regulates angiogenesis (25). Therefore, cancer cells initiate VEGF-mediated angiogenesis to satisfy their increased demand for oxygen and nutrients, resulting in the proliferation of tumor Ecs (26). However, the nascent blood vessels are often structurally and morphologically abnormal and thus fail to adequately supply oxygen and nutrients. This abnormal vasculature also curtails T cell infiltration, whereas the VEGF-mediated pathways prevent the maturation of DC and enhance the recruitment of immunosuppressive cell populations —Treg, M2-TAMs, and MDSC— into the tumor site, resulting in an immunosuppressive TME (26, 27). And, blocking the VEGF-VEGFR axis may promote the accumulation of effector T cells within TME to fire up anti-tumor immunity (28).

#### 2.2.3 Alteration in the three-dimensional stromal environment

Rapidly growing tumors destabilize the structures and functions of surrounding tissues, contributing to structural alterations of the ECM (29). Stromal cells are connective tissue cells of organs and cancer-associated fibroblasts (CAF) are the most common stromal cells in the TME (30, 31). Cancer cells release TGF-β that activates CAF, which then secretes various immunomodulatory chemicals, including IL-11, IL-6, and TGF-β, to not only inhibit anti-tumor immunity but also deposit increasing amounts of ECM (30, 31). Thus, the rigid and dense ECM of the tumor stroma, activated CAF, and pro-fibrotic soluble substances are physical and functional barriers to the infiltration of immune effector cells, ultimately impeding immunotherapy (32).

## 3 Autophagy involved in TME

TME is a dynamic and changing process during cancer development and anti-cancer treatment. Autophagy plays important role in TME remodeling. Hypoxia-induced autophagy in tumor cells or immune cells results in various outcomes, contributing to the formation of the immunosuppressive TME that fuels tumor growth. Autophagy modulation in ECs and stromal cells can affect tumor vascular and three-dimensional stromal environments to regulate immune cell recruitment and function through complicated mechanisms.

### 3.1 Autophagy definition and its related genes

Autophagy is a core intracellular degradation system that transports cytoplasmic components to lysosomes for degradation and renewal —this is a double-edged sword for tumor progression (16). Autophagy suppresses carcinogenesis in the early stages of tumors by removing damaged organelles and DNA. Interestingly, Autophagy is a cytoprotective mechanism for tumors at advanced stages, which increases cancer cell survival and resistance to stresses. This then sustains tumor metabolism, growth, and survival to mediate tumor promotion and development, ultimately promoting tumorigenesis (16). Autophagy is induced by cellular or environmental stress and participates in several intricate biological processes. Initiation, nucleation, elongation, fusion with the lysosome, and destruction are necessary steps of autophagy involving more than thirteen autophagy-related genes (ATG) and proteins (33). Briefly, inhibition of the mammalian target of rapamycin (mTOR) and activation of AMP-activated protein kinase (AMPK) can stimulate the unc-51-like autophagy-activated kinase 1(ULK1) complex, and the class III phosphatidylinositol 3-kinase (PtdIns3K) complex in order, followed by the formation of the phagophore (33). ULK 1 complex includes ULK1, ATG13, FIP200, and ATG101. And PtdIns3K complex contains Beclin1, VPS 34/PIK3C3, VPS 15, ATG 14, and AMBRA-1. In addition, two ubiquitin-like coupling cascades, including autophagy-related 5 (ATG5)-ATG12 and microtubule-associated light chain 3 (MAP-LC3/ATG8/LC3) coupling systems, are required for phagophore elongation. Then, the phagophore grows and fuses on its own to form a double-membrane autophagosome with the LC3-II. And portions of the cytoplasm are gradually engulfed in the autophagosome. Finally, the unions of autophagosomes with lysosomes degrade the cargo and release decomposition products into the cytoplasm for reuse. In addition, substrates can be selectively degraded by various selective autophagy receptors, such as p62/SQSTM1(sequestosome-1) and NBR1. LC3-II acts as a docking site for cargo adaptors that enable cargo loading into autophagosomes (16, 33).

**Figure 2:**
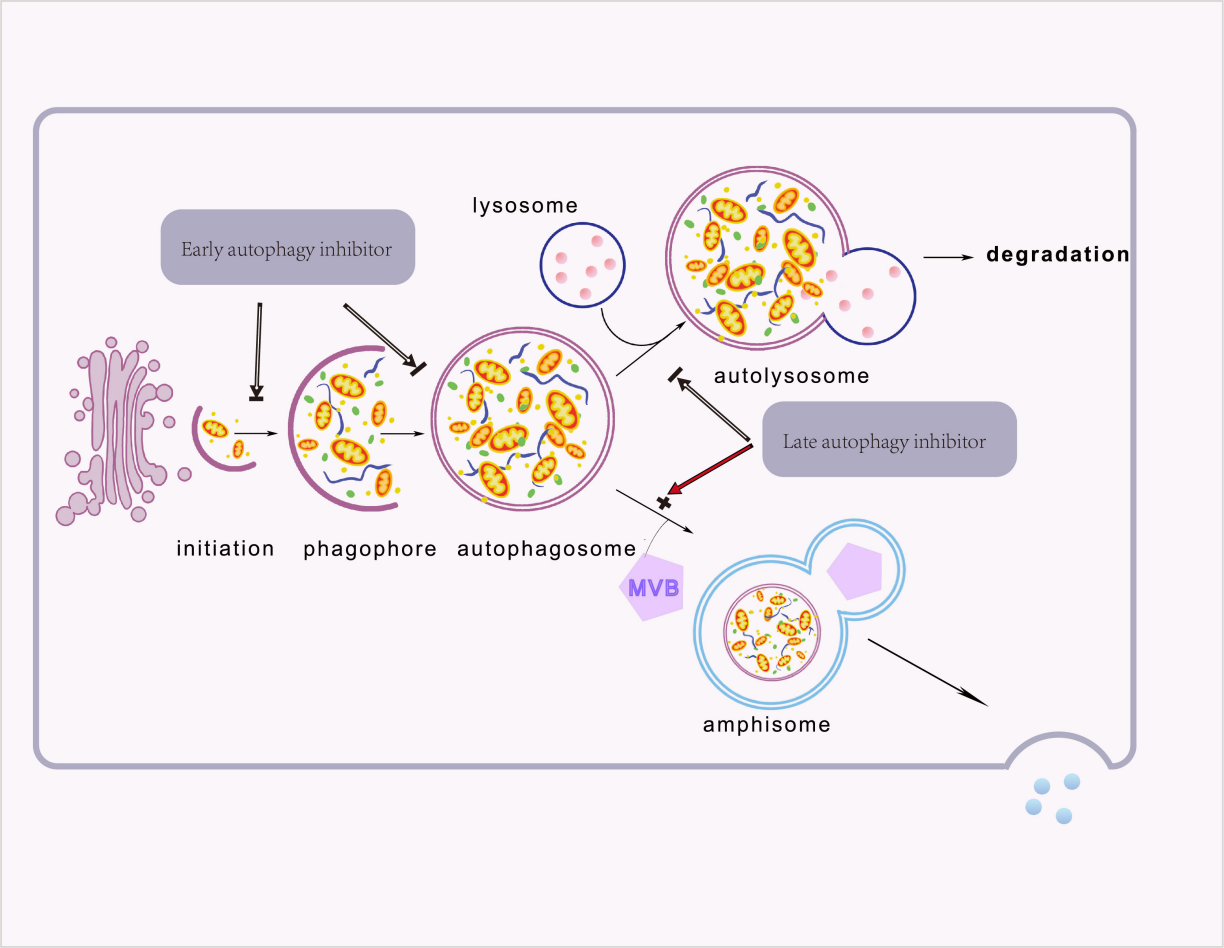
The different types of autophagy. Proteins, organelles, and other cellular components are sequestered in a newly formed isolation membrane. This isolation membrane then swells and seals to form a double membrane-bound vesicle, the autophagosome. Degradation of the autophagosome occurs when the autophagosome fuses with the lysosome. In secretory autophagy, Autophagosomes fuse with multivesicular bodies to produce double-membrane bodies that can fuse with the plasma membrane and secrete cargo into the extracellular space. Furthermore, late and early autophagy inhibitors have different effects on secretion.

Moreover, cancer cells can communicate with neighboring cells *via* secretory autophagy. This autophagy-dependent secretion system affects immune cell function and accelerates tumor growth (34, 35). Although secretory and degradative autophagy both utilize various chemicals and activities (e.g., autophagosome formation, ubiquitin), secretory autophagy does not degrade its cargo *via* lysosomes. Multivesicular bodies and autophagosomes combine to form amphiboles, which then fuse either with secretory lysosomes or directly with the plasma membrane to secrete proteins (34, 36). Autophagosome trafficking depends on outer membrane proteins. For example, LC3-II identifies the secretory and degradative routes, STX17 fuses degradative autophagosomes to lysosomes, and TRIM16 and SEC22B control autophagosome secretion (36). Despite secretory autophagy mediating the secretion of IL-1, IL-8, CXCL6, CXCL8, TGF-β, high-mobility group box 1 (HMGB1), and autophagic vacuoles, its regulation remains unclear (36, 37). These suggest that selectively targeting specific stage of autophagy profoundly affects its accompanied secretory pathways. Furthermore, late and early autophagy inhibitors have contrasting effects on secretion —for example, Spautin-1 and CQ are both autophagy inhibitors but have opposing secretory effects (37). The stage appliance of autophagy inhibitors during the autophagy process was elicited in **Figure 2**.

### 3.2 Autophagy regulates the characteristics of TME

**Figure 3:**
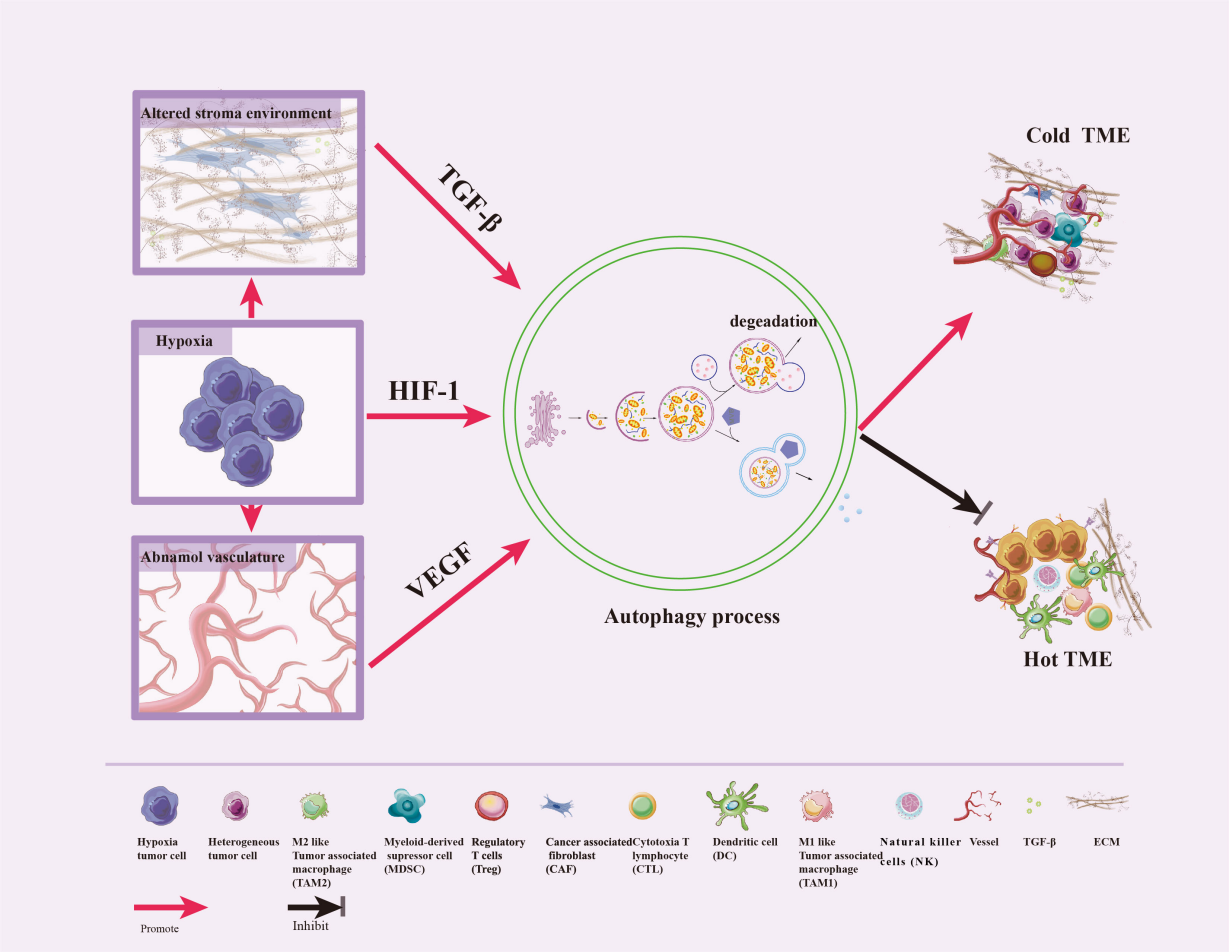
Crosstalk between autophagy and TME features. Hypoxic stress is a typical feature of TME, which triggers the abnormal vasculature and the alteration of the three-dimensional stromal environment. In response to these stresses, autophagy can be induced by HIF-1, VEGF, and TGF-β to promote tumor cell survival, enhance differentiation of normal fibroblasts to CAF, promote dense ECM formation, inhibit immune cells infiltration and vascular normalization, impair CTL and NK cell-mediated anti-tumor immune responses, and convert hot TME to cold.

Hypoxic, alteration in the three-dimensional stromal environment, and abnormal angiogenesis are characteristics of TME, which can induce autophagy in various constituent cells. Autophagy then promotes reshaping of the ECM, remodeling of the cellular composition, and reprogramming of interactions between tumor and stromal cells —this ultimately redefines the TME and thus alters the efficacious of immunotherapies (18, 38). We now review the relationships between some characteristics of TME and autophagy (**Figure 3**).

#### 3.2.1 Autophagy and hypoxia

Hypoxia is the widely accepted stimulator of autophagy induction *via* HIF-1-mediated expression regulation of key genes associated with autophagy, including adenovirus E1B 19 kDa-interacting protein 3 (BNIP3), BNIP3-like (BNIP3L), ATG9A, PIK3C3, Beclin 1, ATG5 and ATG7 (39–43). Increased BNIP3 and BNIP3L expression induced by HIF-1, can disrupt the Bcl-2-Beclin1 complex to initiate autophagy (41). In addition, hypoxia activates the AMP/AMPK/mTOR pathway and inhibits the PI3K/AKT/mTOR pathway to initiate autophagy (44, 45). Through intrinsic cytoprotective pathways, hypoxia-induced autophagy enhances tumor cell survival and contributes to the formation of the immunosuppressive TME that fuels tumor growth (39, 40, 46, 47). For example, HIF-1-dependent autophagy was crucial in inhibiting CTL and NK cell-mediated anti-tumor immune responses (47, 48). In head and neck squamous cell carcinomas, defective autophagy led to increased tumor sensitivity to treatments and lower tolerance to hypoxia (49). In addition, it is noteworthy that autophagy is found to induce HIF-1degradation (50). Further study will be needed to confirm whether autophagy-mediated HIF-1 degradation can consequently affect TME and immune cells. Hypoxia-induced autophagy plays a key role in tumor progression and immunotherapy resistance. How to reduce hypoxia-induced autophagy may be the future research point.

#### 3.2.2 Autophagy and abnormal vasculature

Recent studies demonstrate that autophagy is essential for endothelial differentiation and survival of ECs. Abnormal angiogenesis causes hypoxia, which promotes autophagy in tumor-associated ECs and possibly mediates resistance to hypoxia-induced cell death (51). In addition, autophagy was required for VEGF-mediated endothelial differentiation in breast cancer stem-like cells (52). VEGF treatment activated AMPK-ULK1 axis in breast cancer stem-like cells initiating autophagy (52). In addition, autophagy was a key process for immune sensing and vascular normalization of ECs resulting in governing immune cell recruitment in tumors (53, 54). Autophagy activation in tumor cells may adversely affect numerous angiogenic proteins (such as VEGFR2 and HIF-1) and impede the tumor angiogenic vascular system (55). Soluble decorin, as a partial agonist of VEGFR2, induced autophagic degradation of intracellular VEGFA in ECs to suppress angiogenesis *via* VEGFR2/AMPK/PEG3 axis (56). Administration of autophagic inhibitors such as chloroquine or bafilomycin A1, or depletion of ATG5, results in the accumulation of intracellular VEGFA (56). Furthermore, CQ, an autophagy inhibitor, induces tumor vascular normalization by inhibiting VEGF-A mediated phosphorylation of VEGFR 2 (57). In addition, TME angiogenesis modifies pre-existing vascular characteristics and restricts endogenous T cell migration, impacting both the efficacy and utility of CAR T cell therapy for solid tumors (58). Some studies support that modulation of autophagy in tumor ECs can sensitize immunotherapy. For instance, Endostar prevents angiogenesis by blocking the VEGF-related signaling pathway. In a murine model, anti-PD-1, in combination with Endostar, dramatically enhance PI3K/AKT/mTOR-mediated autophagy, leading to the reversing of immunosuppressive TME (59). And, autophagy in tumor cells impairs T cell survival and function to maintain an immunosuppressive TME. Thus, targeting autophagy may reverse the abnormal vasculature and fire up the immunosuppressive TME, which may enhance the immunotherapy response.

#### 3.2.3 Autophagy affects the three-dimensional stromal environment

Altering the autophagic activity of stromal cells —mainly fibroblasts—, can recreate the three-dimensional stromal environment and induce reprogramming in the TME. Previous reports suggest that autophagy in fibroblasts is upregulated in the TME and plays a role in promoting tumor progression. Firstly, the hypoxic TME induces differentiation of normal fibroblasts into CAF *via* the p62/SQSTM1-autophagy-Nrf2-ATF6 axis (60). Hypoxia-induced TGF-β regulates the activation and function of CAF. And, CAF secretes TGF-β and promotes dense ECM formation, boosting T-cell exclusion *via* the chemical and physical barriers (31). Secondly, autophagy in CAF support pro-fibroproliferative responses, including type 1 collagen deposition and tissue stiffness, resulting in a rigid desmoplastic stroma that impedes CTL infiltration and activation (61). Furthermore, autophagy in CAF regulates immune cell recruitment through the secretion of inflammatory factors such as IL-6 (61). In addition, autophagy activated by TGF-β1 was necessary for the development of myofibroblast and CAF phenotypes, which was associated with enhanced migration and invasion of oral squamous cell carcinomas (62).

## 4 Altered autophagy burns up the immunosuppressive TME to promote anti-tumor immune

**Figure 4:**
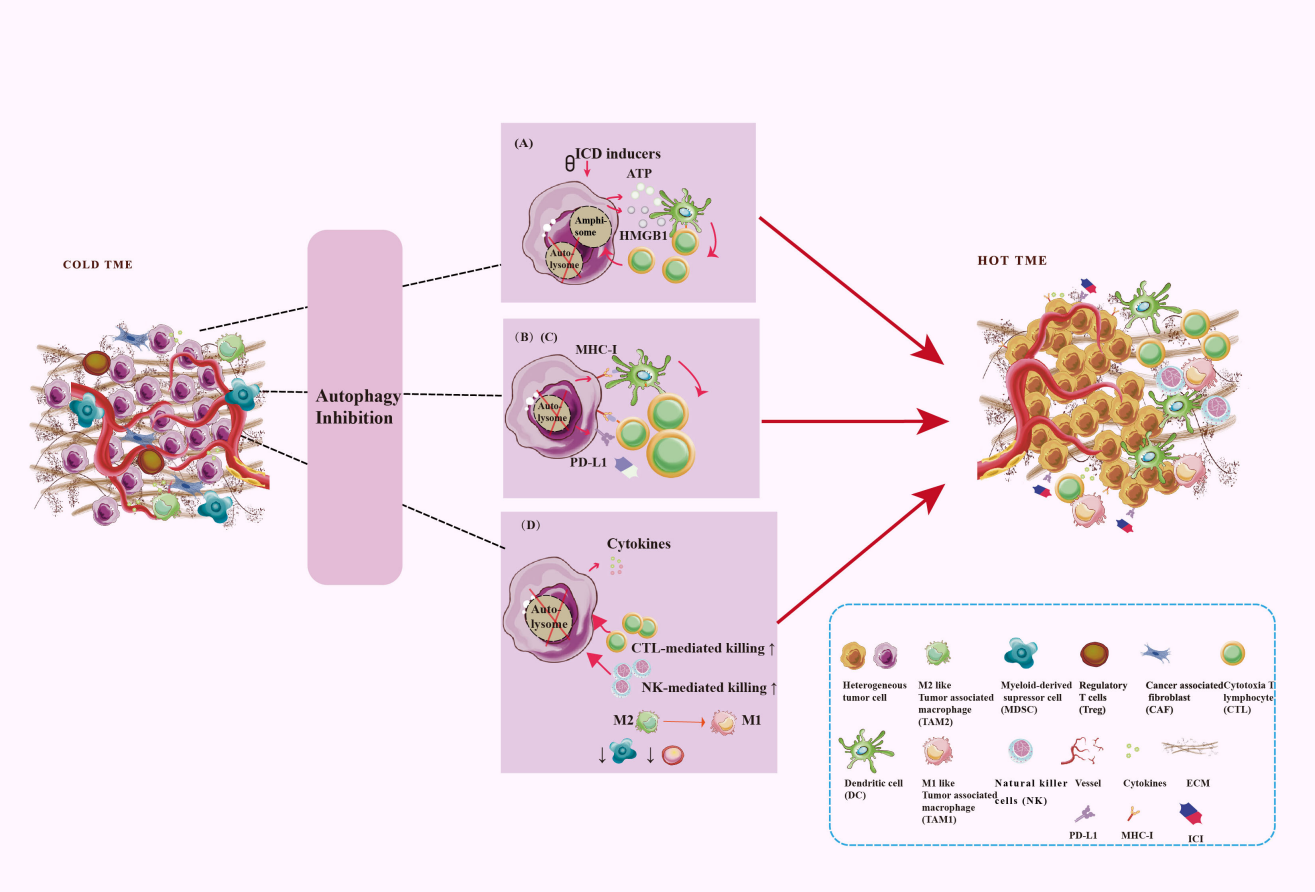
Targeting autophagy burns the cold TME. TME regulation mechanism includes antigen signal release, antigen presentation, antigen recognization, as well as immune cell infiltration. Autophagy plays a key role in these processes. **(A)** Secretory autophagy promotes ICD to burn the cold TME. **(B)** Autophagy inhibits the MHC-I expression on the surface of tumor cells and DC to inhibit antigen presentation. **(C)** Autophagy inhibits PD-L1 expression on the surfaces of tumor cells. **(D)** Autophagy inhibits CD8^+^ T cells and NK cells infiltration and tumor-killing effect and promotes the survival and development of Treg and MDSC. Autophagy promotes TAM polarize to M2 and enhances the immunosuppressive function of macrophages.

Altered autophagy can recreate the immunostimulating TME to promote anti-tumor immune by enhanced immunogenic cell death (ICD), tumor antigen releasement, antigen presentation, promoted antigen recognization, effector immune cells infiltration, and immunosuppressive cell outflow, which may be applied to improve the efficacy of immunotherapy for tumors. With few exceptions, autophagy inhibition is favor influence for burning up “cold” TME. To gain an efficient prospective benefit, we should focus on the targeted cells, targeting autophagy type, effective stages in the autophagy process, and the immune-signal regulatory point of the autophagy modulators. **Figure 4** showed the main process and mechanisms involved in autophagy-mediated TME remodeling. These mechanisms are explicated in **Table 1**.

**Table 1:** The role of autophagy in the transformation of hot TME to cold TME.

Modulation of autophagy	Source of autophagy	The effect of autophagy on immune response	Tumor types	Related mechanisms	Outcome	Ref.
Autophagy induction	Tumor cells	Antigen signal release	Glioblastoma	Secretory autophagy promotes ICD	Immune activation	(63)
		Antigen presentation	Endometrial cancer	Reduce MHC-I expression	Immune inhibition	(64)
		Antigen presentation	Pancreatic ductal adenocarcinoma	Promote MHC-I degradation	Immune inhibition	(65)
		Antigen recognization	Gastric cancer, Bladder cancer,	Reduce PD-L1 expression	Immune activation	(66, 67)
		Antigen recognization	Triple-negative breast cancer, Intrahepatic cholangiocarcinoma, NSCLC	Promote PD-L1 degradation	Immune activation	(68–70)
		Impair CD8^+^ T cell-mediated killing	NSCLC, PyMT-driven mammary tumor	Reduce T-cell recruitment and activation	Immune inhibition	(71, 72)
		Impair CD8^+^ T cell-mediated killing	NANOG+ tumor	Impair sensitivity of NANOG+ tumor cells to T-mediated killing	Immune inhibition	(73)
		Impair NK cell-mediated killing	Melanoma, Breast cancer, Renal cell carcinomas, NSCLC	Impair cancer lysis of NK cells	Immune inhibition	(74–77)
		Impair NK cell-mediated killing	Melanoma	Impair NK cells infiltration	Immune inhibition	(78)
		Enhance MDSC-mediated immune suppression	Triple-negative breast cancer,	Promote MDSC development	Immune inhibition	(79)
		Enhance Treg-mediated immune suppression	NSCLC	Promote Treg infiltration	Immune inhibition	(80)
		Enhance TAM-mediated immune suppression	Multiple murine tumor cell lines	Promote M2-like macrophage polarization	Immune inhibition	(81)
	Macrophages	Enhance TAM-mediated immune suppression	Hepatoma, Glioma, Lung caner	Promote M2-like macrophage polarization	Immune inhibition	(82–84)
		Enhance TAM-mediated immune suppression	Hepatocellular carcinoma	Promote pro-tumor Gal-1 secretion	Immune inhibition	(85)
		Enhance TAM-mediated immune suppression	Colorectal cancer	Inhibit inflammasome	Immune inhibition	(86)
	Treg	Enhance Treg-mediated immune suppression	Colon adenocarcinoma	Enhance lineage and survival of Treg	Immune inhibition	(87)
	MDSC	Enhance MDSC-mediated immune suppression	Breast cancer	Enhance the survival and viability of MDSC	Immune inhibition	(88)
		Enhance MDSC-mediated immune suppression	Melanoma, Breast cancer	Inhibit anti-tumor immunity	Immune inhibition	(89, 90)
Autophagy inhibition	Tumor cells	Impair CTL-mediated tumor-killing	Breast cancer, Triple-negative breast cancer,	Increase immunosuppressive molecular expression on the tumor surface	Immune inhibition	(72, 91)
		Impair NK-mediated killing	Uterine corpus endometrial carcinoma	Downregulate co-stimulatory receptors in NK cell	Immune inhibition	(92)
	CD8^+^ T cells	Impair CTL-mediated killing	Melanoma	Impair CTL proliferation and IFN-γ secretion	Immune inhibition	(93)
	Macrophages	Enhance TAM-mediated immune suppression	Hepatoma	Stimulate the expression of PD-L1	Immune inhibition	(94)

### 4.1 Autophagy regulates immunogenic cell death

By releasing damage-associated molecular patterns (DAMPs) such as HMGB1, calreticulin, and adenosine triphosphate (ATP) into the TME, systemic chemotherapy, localized radiotherapy, and oncolytic viruses enhance the immunogenicity of tumor cells. These DAMPs aid development of DC, activate cytotoxic T cells, produce inflammatory chemokines and cytokines, and polarize TAMs into M1 states, leading to tumor cell death (95). This regulated cell death type is called ICD and contributes to reprogramming immunostimulatory TME (95). Unconventional autophagy−based secretions such as secretory autophagy participated in the release of ICD-related DAMPs, and this could be impaired by ATG5 knockdown (63, 96). Autophagy enables ICD-associated ATP secretion by the preservation of lysosomal ATP pools and secretory autophagy pathway-mediated releases (97). Upon autophagic activation, an ATP-loaded amphisome fused with the plasma membrane *via* the VAMP7-SNARE complex, releasing ATP into the extracellular medium (98). The release of HMGB1 is similar (63). Upon entry into the outer cell membrane, ATP and HMGB1 bind to P2RX7 and TLR4 receptors on the DC (96). However, autophagy also results in antigen damage, which impairs the immune response. DAMPs and pathogen-associated molecular patterns can initiate autophagy, then autophagosomes will eradicate these molecules to maintain immunological homeostasis (99). Based on its role in the phagocytosis of antigens, autophagy is a potential protective mechanism for tumor cells against ICD-induced immune responses (99). HMGB1 secretion was inhibited by early autophagy inhibitors. Conversely, the late autophagy inhibitors CQ and Bafilomycin A1 increased HMGB1 secretion (37). Therefore, a current research focus is on how to regulate this pathway to attenuate degradative autophagy and enhance secretory autophagy, thereby promoting ICD and igniting cold TMEs. Combining late autophagy inhibitors with ICD inducers to potentiate cancer immunotherapy is a current innovative strategy.

### 4.2 Autophagy regulates tumor antigen releasement

Autophagy degrades proteins of tumor cells such as tumor-specific and tumor-associated antigens. When proteasomes and lysosomes are both inhibited, SLiPs, DRiPs, and misfolded proteins form protein aggregates (ALIS/aggresomes), activating autophagy *via* interactions between p62/SQSTM1 and ATG8/LC3. Peptide intermediates linked to HSP90 are in autophagosomes and through secretory autophagy, they are secreted from tumor cells as cross-presenting immunogenic substrates. These tumor cell-derived autophagosomes are called Defective Ribosomal Products-containing Blebs (DRibbles). As demonstrated in clinical and experimental models, DRibbles are efficient carriers of tumor antigens that induced robust cross-presentation by APCs (100, 101). DRibbles-pulsed-bone marrow cells or DC were peri-tumorally titrated with GITR agonists and PD-1 blocking antibodies, increasing the cytotoxicity activity of CD8^+^ T cells *via* an antigen-presenting independent mechanism (102). In addition, tumor cell-released autophagosomes (TRAPs) converted macrophages to an immunosuppressive M2-like phenotype that is defined by PD-L1 and IL-10 expression, resulting in T cell inactivation and cold TME formation (81). Thus, secretory autophagy plays a key role in tumor antigen releasement, evoking the CD8^+^ T cells priming.

### 4.3 Autophagy impairs antigen presentation

Endogenous antigens of tumor cells are presented to the anti-tumor immune response in two distinct ways. First, the antigens are directly presented by MHC-I of the tumor cells for recognition and elimination by CD8^+^ T cells. Second, DC recognize tumor antigens *via* pattern recognition receptors, process them, and cross-present antigens to T cells, resulting in the activation of anti-tumor CD8^+^ T-cell immunity (7). Some researches demonstrate the role of autophagy in antigen presentation. Autophagosome membranes are possibly produced by the endoplasmic reticulum, and thus peptide-sensitive MHC class I molecules may be present therein (7). Furthermore, autophagy inhibition possibly enhances MHC-I expression on the surface of DC and tumor cells for reducing endocytosis and degradative function, thereby promoting activation and migration of DC, and attraction of CD8^+^ T cells. Ultimately, these ignite the immunosuppressive TME and overcome resistance to the immune checkpoint blockade (ICB) (99, 103). Inhibiting the selective autophagy of MHC-I —this was mediated by the autophagic cargo receptor NBR1— increased MHC-I expression in tumor cells (65). In endometrial cancer, overexpression of LC3 constrained the MHC-I transactivator nucleotide-binding oligomerization domain-like receptor family caspase-containing recruitment domain 5 (NLRC5), a transcriptional regulator of the MHC-I gene, which then decreased the expression of MHC-I (99). Blocking tumor-derived progranulin destroys autophagosomes, restoring MHC-I expression. Then, progranulin antibody therapy increases levels of granzyme B, TNF, IFN-γ, and CD8^+^ T cells, reviving CD8^+^ T cell anti-tumor cytotoxicity (64). Adaptor-associated kinase 1 (AAK1) mediated endocytosis and autophagic degradation of MHC-1 in DC, leading to inhibition of antigen presentation and CD8^+^ T cell initiation. However, DC with ATG5/ATG7 deficiency showed increased MHC-I expression (103). DC are the most efficient specialized APCs. DC may store antigen in endolysosomal compartments for extended periods, and this preserves MHC-I antigen cross-presentation to CD8^+^ T cells. However, autophagic processes compromise DC antigen storage and presentation. For example, DC from ATG5-deficient mice stored antigen in storage compartments for prolonged periods, consequently curtailing late MHC-I cross-presentation and boosting antigen cross-presentation into CD8^+^ T cells (104). DC from VPS34-deficient animals had homeostatic maintenance along with a partially activated phenotype, spontaneously generated cytokines, and displayed the increased activity of conventional MHC class I and class II antigen-presentation pathways (105). Autophagy inhibition combined with anti-PD-1 therapy increase tumor neoantigen presentation in LKB1-inactivated high-TMB tumor and achieve a greater anti-tumor effect (106). Thus, autophagy inhibition may enhance antigen presentation through elevated MHC-1 expression and activated DC phenotypes, leading to the priming of T cells and enhancement of the immunotherapy effect.

### 4.4 Autophagy promotes antigen recognization

Immune checkpoints are pathways with inhibitory or stimulatory properties, modifying immune cell activity. The most well-known inhibitory checkpoints are PD-1 and PD-L1, which inhibit T cell activation, proliferation, and function (107). ICIs are a breakthrough in treating tumors and applied to block the binding between PD-L1 and PD-1, which can re-activate CTLs and NK cells (107). Links between autophagy and immune checkpoints have recently been established. For example, ROS generated by andrographolide inhibits the JAK2/STAT3 pathway in NSCLC, leading to the activation of p62-dependent selective autophagy and promoting the degradation of PD-L1 (68). Sigma1 is a ligand-operated integral membrane chaperone —scaffolding protein— that is abundant in endoplasmic reticulums of various cancer cell lines and generally binds to glycosylated PD-L1 to preserve protein stability (69). Pharmacological suppression or deletion of the Sigma1 decreases PD-L1 expression on the tumor surface *via* selective autophagy (69). KRAS-mutated intrahepatic cholangiocarcinoma cells had activated ERK signaling. Blocking of ERK signaling induced autophagy to degrade PD-L1, while genetically silencing the ATG7 expression partially reversed degradation (70). Furthermore, ATG7 overexpression enhanced the steadiness and expression of PD-L1 mRNA by autophagy-induced FOXO3a/miR-145 degradation in bladder cancer models (66). In gastric cancer cells, inhibiting autophagy causes the buildup of p62/SQSTM1 and the activation of nuclear factor (NF)- κB, resulting in the induction of PD-L1 (67). Controversially, another study showed that 5-HT activated p-STAT3/autophagy axis *via* 5-HT1aR, leading to upregulated PD-L1 expression and an immunosuppressive environment, which remains to be confirmed in the further steps (108). Taken together, autophagy modulators may be a new immunosensitizer, for it directly or indirectly downregulates PD-L1 expression.

Another newly discovered immunological checkpoint CD155 expressing on tumor cells functions as a ligand for the costimulatory receptor CD226 and the co-inhibitory receptor TIGIT of natural killer cells and T cells. ATG5-dependent autophagy induced by artesunate could enhance CD155 overexpression on uterine somatic endometrial carcinoma (UCEC) cells. CD155 overexpression upregulated CD226 and downregulated TIGIT, hence enhancing the cytotoxicity of NK cells (109). However, the precise mechanism is unknown. Other immune checkpoints SIRPα and CD47 release a “don’t eat me” signal to prevent the recognization and phagocytosis of cancer cells by immune cells. The interaction of CD47 on cancer cells with SIRPα on macrophages could suppress the phagocytosis of macrophages (110). By blocking the CD47/SIRPα axis, SIRPαD1-Fc selectively targets NSCLC cells and activated macrophages to recognize and phagocytose tumor cells (110). But it also causes protective autophagy in cancer cells, which may be a reaction to cellular stress. Concurrently targeting CD47 and autophagy improves macrophage-mediated phagocytosis and cytotoxicity against NSCLC cells (110). Zhang et al. discovered that autophagy in glioblastoma cells could impair the immunotherapeutic benefits of anti-CD47-SIRPα therapy through decreased phagocytosis of macrophages and decreased cytotoxicity of CD8^+^ T cells (111).

Thus, autophagy regulates antigen recognization mainly by the degradation of immune checkpoint proteins and the regulation of intracellular signaling pathways thereby influencing antitumor immune responses and immunotherapy efficacy. Combining autophagy modulators with ICIs may be a promising anti-tumor therapy to improve the effectiveness of ICIs and need to be further explored in clinical application.

### 4.5 Autophagy regulates the recruitment of immune cells

#### 4.5.1 Autophagy regulates CTL- and NK- mediated tumor killing

Tumor cell eradication depends on the attraction and subsequent infiltration of CTLs and NK cells into the TME, the absence of which contributes to immunotherapy resistance and tumor progression (38). Relatedly, autophagy affects CTL activity and infiltration (112). For example, FIP200, an essential autophagy gene, is responsible for restricting T cell recruitment and activation in the TBK1/IRF/IFN signaling axis. In immunocompetent breast cancer models, impairment of this noncanonical autophagic function of FIP200, in combination with immune checkpoint blockade therapy, yielded good responses (71). SKIL promoted the growth of tumors and prevented the entry of CD8^+^ T cells into NSCLC cells by upregulating the TAZ/autophagy axis and downregulating the STING pathway (72). In addition, alteration of autophagy can increase tumor cell sensitivity to T cell-mediated tumor killing. For instance, NANOG was a major transcription factor that enhances secretory autophagy in tumor cells *via* promoting LC3B expression, leading to EGF autocrine (73). EGF subsequently upregulated the EGFR-AKT signaling pathway and then led to tumor cell resistance to CTL killing (73). Early autophagy inhibitors combined with ICI can reverse tumor refractory phenotype (73). However, ATG7/ATG5 deficient triple-negative breast cancer cells were less vulnerable to T cell-mediated death due to the p62-mediated selective autophagy of Tenascin-C, a candidate immunosuppressor (113). Similarily, B7H3 expressed on tumor cell surface inhibited not only activation and proliferation of T cells but also the production of immunostimulation cytokine (114). And, autophagy has been affirmed to participate in the degradation of B3H7 (91). In breast cancer models, V9302 decreased B7H3 expression and increases CD8^+^ T cell activation by the autophagy-lysosome pathway (91). In addition, anti-PD-1/PD-L1 mAb combined with B7H3 blockers (anti-B7H3 mAb or V9302) could transform “immune desert” tumors into “hot”, improve the curative benefits in metastatic or advanced breast cancer (91). Furthermore, autophagy is also involved in regulating T-cell immune activity. ATG3^-^, ATG7^-^, or ATG5^-^ T cells cannot proliferate efficiently (115). DeVorkin et al. showed that loss of autophagy triggered T cells into a glycolytic phenotype along with reduced S-adenosylmethionine levels. Thus, ATG5^-/-^ CD8^+^T cells gained an effector memory state that promoted CD8^+^ T cell-mediated tumor rejection and INF-γ release (116). In brief, autophagy plays a “double-edged” regulation in CTL cell-dependent immunotherapy. It is required to consider that autophagy modulators affect which target cells in the combination of immunotherapy.

NK cells express a range of stimulatory and inhibitory receptors that determine whether to kill tumor cells by binding to specific ligands on tumor cells (9). Autophagy involves the cytolytic activities, and memory responses of NK cells and essentially participates in the downregulation and activation of effector molecules and receptors respectively (117). Activated NK cells form synapses with the tumor cells and release two cytotoxic effectors (perforin and granzyme) to mediate the death of tumor cells. The gap-junctional connexin 43 is required for the synapse (74). Hypoxia-induced connexin 43 overexpression could selectively induce autophagy to hinder its localization on the immunological synapse to promote tumor cells’ evasion of NK cell-mediated death. However, ATG5 siRNA-mediated autophagy inhibition can reverse these processes (74). Moreover, connexin 43 channel is also an essential component of CTL cytotoxic immunosynaptic-mediated tumor cell death (118). In addition, autophagy selectively destroys the NK-derived granzyme B in cancer cells, reducing tumor cell sensitivity to natural killer-mediated lysis. Targeting BECN1/Beclin1 and ULK1 can revive the cytotoxicity of granzyme B (75–77). Likewise, the dysfunctional autophagy of cancer cells enhances the recruitment of NK cells to the tumor periphery. Becn1/Beclin1-deficient melanoma cells in the TME expressed high levels of CCL5 *via* the activation of the MAPK8/JNK-JUN/c-Jun signaling pathway, which boosted the infiltration of functional NK cells into the TME and curtailed tumor progression (119). Furthermore, when autophagy was inhibited in melanoma cells by either ATG5 or p62/SQSTM1 deficiency or CQ treatment, melanoma cells could recruit NK cells into the tumor site by CCL5 releasing (119). Blocking autophagy of tumor cells promote NK cell-mediated aggregation and killing, converting TMEs from cold to hot. Autophagy inhibitors possibly potentiate CTL-based or NK cell-based immunotherapies.

#### 4.5.2 Autophagy promotes the recruitment and function of immunosuppressive cells

Malignant tumors recruit immunosuppressive cells —such as TAM with an anti-inflammatory M2 phenotype, MDSCs, and Tregs— to suppress T cell functions and form the immunosuppressive TME. Conversely, effective T and NK cells are rare in “cold” TME. Autophagy essentially contributes to the activation and infiltration of these immunosuppressive cells, which may impair T-mediated tumor killing, reshaping the TME into a cold state and ultimately causing resistance to immunotherapy (92).

Tumor cells can actively attract circulating monocytes to the tumor site by secreting CCL2, and then the recruited monocytes developed into TAMs through granulocyte-macrophage colony-stimulating factor (GM-CSF) and macrophage colony-stimulating factor (MCSF) (120, 121). These chemokines and cytokines induced autophagy of monocytes to keep them alive and allow them to differentiate into TAM (120, 121). In response to stimuli from TMEs, various TAMs are polarized to M2-like phenotypes and this results in immunosuppression and tumor progression. Macrophages took up Beclin1-dependent tumor cell-released autophagosomes and activated MyD88-p38-STAT3 axis *via* TLR4. This resulted in overexpression of PD-L1 and IL-10, which limited CD8^+^ T cell recruitment and promoted tumor formation (81). In addition, hepatocellular carcinoma-derived HMGB1 triggered M2 macrophage polarization *via* a TLR2/NOX2/autophagy axis (82, 83). Furthermore, Inhibition of autophagy in macrophages reprogrammed pro-tumor M2-like TAMs to a tumor-suppressing M1 phenotype that exerted anti-tumor effects by regulating not only NF-κB p65 protein homeostasis but also an IL-6-pSTAT3-miR-155-3p-autophagy-pSTAT3 positive feedback loop (82, 83). In addition, Galectin-1 was a soluble tumor-promoting factor secreted by TAMs through TLR2-dependent secretory autophagy, —it was associated with poor outcomes (85). However, a recent study suggested that autophagy invalidation in macrophages induces an immunosuppressive phenotype to alter the antitumoral immune response, leading to hepatocarcinogenesis (94). The phagocytosis of TAMs is efficient in the early stages of tumor-specific antigen processing and innate tumor killing. Autophagy is a requirement for TAMs phagocytosis (122). Through IDO1 expression and kynurenine metabolism, IFN-γ promoted autophagy and macrophage phagocytosis in cervical cancer cells (122). In summary, autophagy functions as a regulatory process in macrophages, maintaining cellular homeostasis and regulating specific immune functions such as recruitment, differentiation, polarization, phagocytosis, and pro-tumor factors production. Targeting autophagy in macrophages may thus be a novel and practical anti-cancer approach.

Tumor-infiltrating Tregs suppress anti-tumor immune responses and promote tumor immune escape (11). Autophagy in tumor cells linked to Treg infiltration and immunosuppressive activities of TME. Results from the NSCLC xenograft model showed that tissue-specific knockdown of ATG5 attracted Treg migration to the TME (80). Except for this, the autophagy pathway also participated in Treg cell lineage differentiation and function. As the transcription factor FOXP3 is required for the differentiation and immunosuppressive activity of Treg cells, Treg cells with the autophagy-related genes AMBRA1 and ATG7 deletion fail to express FOXP3, leading to the malfunction of Treg cells (123). In addition, ATG5^-^/ATG7^-^ Treg cells displayed functional deficiency *via* increasing mTORC1 expression, c-Myc expression, and glycolytic activity (87).

MDSCs are immunosuppressive cells derived from bone marrow progenitor cells and immature bone marrow cells (12). To meet their bioenergetics and biosynthesis requirements, tumor cells reroute metabolic pathways one of which, is glycolysis, a hallmark of cancer (79). Glycolysis enhanced granulocyte colony-stimulating factor (G-CSF) and granulocyte-macrophage colony-stimulating factor (GM-CSF) expression *via* the AMPK-ULK1 and autophagic pathways, which then promote MDSC development (79). MDSCs activate autophagy to survive in the harsh TME induced by cellular stresses such as nutrition and hypoxia. Under these stressful conditions, MDSCs released HMGB1 to maintain their viability *via* initiating autophagy (88). Autophagy-deficient MDSCs displayed reduced lysosomal degradation, which promoted the surface expression of MHC class II molecules and resulted in the effective activation of tumor-specific CD4^+^ T cells (89). In addition, tumor growth increased β2-AR expression in MDSC leading to enhanced autophagy and activation of arachidonic acid *via* pressure-activated signals. These increased the release of PGE2, an immunosuppressive mediator (90). Taken together, autophagy promotes the immunosuppressive function of MDSC, thereby facilitating the formation of an immunosuppressive TME.

## 5 Autophagy-targeting strategy assists in immunotherapy

The effectiveness of immunotherapy is significantly influenced by the cold TME, which is also regulated by autophagy. Therefore, strategies targeting autophagy should be exploited to develop efficient immunotherapy sensitizers. However, the physiological processes involved in autophagy are complex and there is no consensus on whether autophagy should be activated or suppressed. Almost all stages of autophagy, including vesicle nucleation, maturation, fusion, and lysosomal destruction, have been identified as potential therapeutic targets. Understanding these regulatory pathways may lead to the development of new cancer treatment options.

### 5.1 Targeting autophagy

Autophagy acts as a guardian to promote tumor cell survival in response to stress in the TME. Nowadays, various autophagy inhibitors are accessible drugs being applied to target autophagy, being cataloged as early-stage inhibitors (SAR405, 3MA, and SBI-0206965) and late-stage inhibitors (CQ, HCQ) (124). Early-stage inhibitors regulate the nucleation of the autophagy process by targeting ULK1/ULK2 or VPS34, while late-stage inhibitors target lysosome disorder the degradation stage of the autophagy process (124). So far, secretory autophagy-based clinical studies are in the preliminary stage, and late and early-stage inhibitors of autophagy have diverse effects on secretory autophagy (37). In addition, mTOR inhibitors also play an important role in antitumor therapy as enhancers of autophagy initiation (125). Autophagy targeting compounds used for cancer treatment are listed in **Table 2**.

**Table 2:** The applied autophagy targeting agents in treatments *in vivo* and *vitro*.

Compound	Molecular Target	Stage of autophagy	Type of cancer	Effect on autophagy	Outcomes	Ref.
SBI-0206965/MRT67307	ULK1/2	Initiation	Triple-negative breast cancer	Inhibition	Reduce the viability of triple-negative breast cancer cells	(126)
SAR405	PIK3C3/Vps34	Nucleation	Melanoma and Colorectal cancer tumors	Inhibition	Induce an infiltration of NK, CD8^+^ T cells, and CD4^+^ T cells	(127)
3-MA	PIK3C3/Vps34	Nucleation	Colon carcinoma	Inhibition	Increase tumor cell sensitivity to chemotherapy	(128)
CQ	Lysosome	Fusion	Melanoma	Inhibition	Remodel TMA toward the M1 phenotype	(129)
HCQ	Lysosome	Fusion	Gastric cancer	Inhibition	Remodel blood vessel	(130)
GNS561	PPT1	Degradation	Hepatocellular carcinoma	Inhibition	Promote tumor cell death	(131)
Rapamycin	MiR-26a-5p/DAPK1	Initiation	Glioma	Activation	Have anti-tumor effects	(125)
ABTL0812	Akt/mTOR	Initiation	Pancreatic cancer	Activation	Transform cold tumors into hot tumors	(132)

Evidence from preclinical studies suggests that targeting autophagy can improve the efficacy of numerous cancer treatments. Several US Food and Drug Administration (FDA)-approved medications, including the inhibitor CQ and its derivative HCQ, as well as inducers like rapamycin, have been identified as effective autophagy modulators (125, 130). A previous meta-analysis reported that autophagy inhibitors, such as CQ and HCQ alone or combined with other anti-cancer drugs were well tolerant and significantly improved cancer patients’ overall response (133). Rapamycin exerts anti-tumor effects by promoting autophagy in glioma cells, which was dependent on the miR-26a-5p/DAPK1 pathway activation (125). Although many small molecule therapeutics have been identified as effective anti-cancer drugs, further clinical investigations are being carried out. The compound GNS561 inhibits the enzyme palmitoyl protein thioesterase 1 (PPT1), causing Zn^2+^ accumulation in the lysosomes and reducing autophagic flux. Therefore, it is regarded as a promising anti-cancer therapy (131). By activating cytotoxic autophagy in tumor cells, the autophagy inducer ABTL0812 increases the death of cancerous cells. The first human Phase I/Ib dose-escalation clinical trial demonstrated that ABTL0812 was safe, tolerable, and has powerful anti-cancer properties (134).

### 5.2 Combination in targeting autophagy and TME components for immunotherapy

Immunotherapy is widely used to treat advanced tumor patients. However, the efficacy of immunotherapy is impeded by cold TME. Autophagy regulates intracellular homeostasis, cell survival, cell activation, cell proliferation, and differentiation. Therefore, modulating autophagy can remodel the immunosuppressive TME into immunostimulatory TME. The accumulated evidence of clinical trials demonstrated that an autophagy-targeting strategy can improve the efficacy of immunotherapy. **Table 3** describes the ongoing trials about the combination of autophagy inhibitors and immunotherapy for cancer treatment registered on ClinicalTrials.gov (https://clinicaltrials.gov/). Due to their positive effects on tumor cells and animal models, PD-L1 inhibitors and autophagy inhibitors CQ are the focus of most ongoing clinical trials. Other combination therapies including TME targeting are also being investigated in preclinical studies, and the results are encouraging.

**Table 3:** The ongoing clinical trials about the combinations of immunotherapy with autophagy targeted drugs.

Clinical trial identifier	Treatment	Cancer types	Phase
NCT03057340	Dribble vaccine	NSCLC	I
NCT04841148	Avelumab/Hydroxychloroquine + Palbociclib	Breast Cancer	II
NCT04214418	Cobimetinib + HCQ + Atezolizumab	KRAS-mutated Advanced cancer	I/II
NCT04787991	HCQ/Nivolumab + Ipilimumab + nP/gem	Metastatic Pancreatic adenocarcinoma	I/II
NCT04464759	Nivolumab/Ipilimumab + HCQ	Advanced Melanoma	I/II
NCT03344172	HCQ + Gemcitabine + Nab-paclitaxel + avelumab	Pancreatic cancer	II
NCT01550367	HCQ + Aldesleukin (IL-2)	Metastatic renal cell carcinoma	I

#### 5.2.1 Targeting autophagy to enhance ICD

In recent research, researchers have focused on developing strategies to increase autophagy to improve ICD effects. Local administration of low-dose chemotherapeutic medicines plus the autophagy enhancer rapamycin (CAER) led to systemic anti-tumor T-cell immunity *in vivo*. In addition to enhancing the mortality of B16F10 and 4T1 tumor cells and increasing the levels of autophagy *in vitro*, the low-dose CAER treatment promoted neoantigen-specific T-cell responses. It also modified the TME by lowering systemic toxicity (135). In MDA-MB-231 and CT26 cancer cells, brucine enhanced the effects of ICD, such as CRT exposure and HMGB1 release, and inhibited autophagy by hindering the destruction of autolysosomes. However, ATG5 knockdown significantly decreased the release of HMGB1 and CRT exposure caused by brucine (136). And, early autophagy inhibition can reduce ICD but late autophagy inhibition can increase it, which is dependent on the secretory autophagy response generated by the autophagy inhibitor (137). In contrast, an optimal dose of combined LipSHK (ICD inducer) and LipHCQa (autophagy inhibitor) could maximize ICD-based antitumor immunity in colon cancer (138). Thus, combining ICD inducers with autophagy targeting drugs may be an innovative strategy to improve cancer immunotherapy. Moreover, the secretion of HMGB1 and ATP may be effective markers for treatment response prediction.

#### 5.2.2 Targeting autophagy to assist immune checkpoints inhibitors

The absence of tumor-infiltrating T cells and recruitment of diverse immunosuppressive cells are the significant features of “cold” TME, causing resistance to ICls. And, targeting autophagy has been found to regulate anti-tumor immune response by remodeling the immune TME. Therefore, combination treatment approaches may enhance the response to ICIs in cancer patients. For example, the autophagic cargo receptor NBR1 plays a key role in the targeting of MHC-I molecules for lysosomal degradation. In pancreatic ductal adenocarcinoma cells, MHC-I is less visible on the cell surface but is more pronounced in autophagosomes and lysosomes. CQ and dual ICB treatment (anti-PD1 and anti-CTLA4 antibodies) synergize to enhance the immune system’s ability to fight tumors (139). Researchers demonstrated that combined SIRPα-Fc and CQ treatment interrupted the CD47/SIRPα axis and disrupted the protective autophagy in tumor cells, enhanced phagocytosis of macrophages, and then activated CD8^+^ T cell-mediated anti-tumor immune (111). In addition, HCQ and rapamycin treatment reduced autophagic flux and expression levels of CD47 and SIRPα, thereby enhancing the phagocytosis of TAM, which has strong phagocytic activity (140). HCQ and rapamycin treatment could also improve anti-PD-1 therapy by reprogramming M2-like TAM to M1-like phenotype and enhancing T cell-mediated cytotoxicity (141). Combined with the anti-PD-1 therapy, CQ/HCQ targeting palmitoyl protein thioesterase 1 (PPT1), a novel regulator of cancer cell autophagy, enhances the anti-tumor immune response by switching the macrophage M2 to M1 phenotype, lowering MDSCs, and increasing T cell-mediated cytotoxicity (142). In addition, silencing the autophagy-associated protein BECLIN1 or VPS34 promotes the release of pro-inflammatory CCL5 and CXCL10 in the TME of melanoma and CRC tumor cells *via* activation of STAT1/IRF7 axis, resulting in increased infiltration of central immune effector cells (NK, CD8^+^ and CD4^+^ T cells, DC, and M1 macrophages). And, SB02024 or SAR405 (VPS34 inhibitors) converts cold immune deserts to hot immune TMEs and reverses anti-PD-1/PD-L1 treatment resistance in melanoma and CRC tumor models (78, 143). ESK981, a novel autophagy inhibitor, was identified to increase the susceptibility of cold tumors to ICIs by producing CXC10 that draws T cells by targeting the autophagy-associated protein Pikfyve in prostate cancer cells (144, 145). Although Pemetrexed and cisplatin (PEM/CDDP) chemotherapy combined with ICIs did not have the synergic effect in patients with metastatic NSCLC, the combination regimen with MEK inhibitors (MEKi) blocking autophagy could trigger the CXCL10 secretion and CD8^+^ T cell recruitment to enhance the tumor-killing effect (146). Furthermore, in LKB1 mutant tumor models, ULK1 suppression and PD-1 antibody inhibition act as co-promoters of the effector T cell growth and tumor regression. Mechanistically, LKB1 deficiency inhibits the production of immune peptides by reducing the expression of the immunoproteasome component. This state can be changed by inhibiting the autophagy regulator ULK1, which increases the level of immunoproteasome expression and then increases lung tumor infiltration in CD4^+^ and CD8^+^ T cells (106). By modulation of immune recognition, immune effector cell chemotaxis, immune suppressor cell reduction, and antigen presentation in the TME, autophagy inhibitors can increase the infiltration of CTL cells in the TME, contributing to enhancing ICIs treatment.

#### 5.2.3 Targeting autophagy is a potential adjuvant of CAR-agents

Currently, the most advanced forms of cancer immunotherapy are CAR-T cell therapy. Immunotherapy known as CAR T cells uses T cells taken from patients, and genetically altered to express receptors that identify cancer-specific antigens, and then transfused (147). Due to the hostile solid TME, which acts as a barrier to CAR T cell infiltration and activity, CAR T cell treatment has been clinically successful in treating hematologic tumors but unsuccessful in treating solid malignancy (147). Because autophagy can effectively control the immunosuppressive TME, it may be beneficial in patients with solid tumors receiving CAR T cells. In mice with gliomas, autophagy significantly alerts the persistence of CAR-T and acts as an antagonist to CAR-induced trogocytosis and immune checkpoint activation (148). This suggests that autophagy stimulation may promote CAR T cell tumor fitness and survival in TME (148).

Because of the elevated levels of the tumor-secreting chemokines CCL5 and CXCL10, autophagy inhibition increases the formation of NK cells in glioblastoma *in vivo*. Furthermore, suppression of autophagy alters NK cell phenotypes to improve NK cell function and promotes NK cell-mediated cytotoxicity against glioblastoma cells. In one study, the combination of CQ and multifunctional genetically engineered NK cells outperformed multifunctional genetically engineered NK cells alone, inhibiting the growth of GBM tumors (149).

#### 5.2.4 Autophagy and vaccine

Some cancer vaccines have been suggested to be effective immunotherapy for several malignancies, including glioma, breast cancer, and liver cancer. They deliver high-quality antigens to active APCs and induce strong CD4^+^ T helper cell and cytotoxic T lymphocyte responses (150). Inducing autophagy in DC promotes peptide presentation to CD4 T cells, which is a novel strategy for increasing vaccine efficacy (151). Professional APCs transmit tumor proteins to T lymphocytes for activation *via* the MHC-I after consuming and degrading them with proteases during cross-presentation. Defective ribosome products (DRiPs) and short-lived proteins (SLiPs), two putative tumor-associated proteins, are produced in large amounts by tumor cells but are intrinsically uns` and only momentarily expressed under physiological conditions before being polyubiquitinated and broken down by tumor cell proteasomes. Inhibiting proteasomal degradation and altering the cellular autophagic pathway results in the production of the DRibbles vaccine product, which stabilizes the DRiPs/SLiPs proteins and induces the formation of autophagosomes that contain the proteins mentioned above as well as other protein products that are known to facilitate cross-presentation (152). A previous study use proteasome and lysosome inhibitors to prepare CMV-autophagosomes (DRibbles) (150). They show that IFN-DC loaded with DRibbles activated CMV-specific T cells (150).

## 6 Conclusions and future perspectives

It has been acknowledged that by boosting the anti-tumor immune response, immunotherapy improves the prognosis of patients with advanced cancer. However, only a tiny percentage of individuals with advanced disease have a satisfactory and sustained response to immunotherapy. Immunotherapy fosters further development of the TME. All TME components either negatively or positively influence the response to immunotherapy. By regulating antigen release, antigen presentation, antigen recognition, and immune cell trafficking, targeted autophagy therapy can create hot TME to improve the efficacy of cancer immunotherapy. In addition, immunological characteristics of TME are important factors to be considered in the strategies for immunotherapies improvement.

Even though several methods have been developed to influence the immune system and improve clinical outcomes, further investigation is needed to determine the mechanisms involved. Although few clinical studies have been conducted, several cellular and animal experiments combining autophagy targeting with immunotherapy have been conducted. Even though the majority of cellular and animal trial outcomes demonstrate that combination therapies are superior to immunotherapy alone, it is not clear whether the efficacy is similar across species. Notably, only CQ or HCQ are used as autophagy inhibitors in cancer therapy. However, their therapeutic outcomes are limited by their high toxicity and poor selectivity. Consequently, further research should be carried out to explore the target genes associated with autophagy. From a clinician’s standpoint, autophagy-targeted medicines should be customized to treat specific stages and grades of cancer.

Currently, there is no consensus on the best time to administer autophagy-targeted medications—before, during, or after immunotherapy. Autophagy-targeting drugs can alter the tumor’s immune milieu, hence influencing immunotherapy. To promote the effectiveness of immune-oncology medications, effective dosing schedules of autophagy modulators alone or in combination should be explored to modify the immunologic activity of TME in time and make a balance between side effects and tumor response benefits. Moreover, research is needed to determine multiple modality-sensitive indicators for predicting the patients’ future responses to anti-tumor alone or combination therapies. For improving clinical appliance, we can also focus on the affecting process, stage, and sequence of autophagy in TME remodeling, and how to conduct precise modulation of autophagy in the appropriate target cells of TME. Although suppressing tumors by targeting autophagy is currently thought to be a good therapeutic strategy, further research is required to determine how the host will be affected in the long run. Future studies are also needed to explain the “occasionality” interaction between autophagy modulators and immunological response. Additionally, the distinct cell populations that control autophagy and hence remodel the immune milieu should be explored, as well as whether the association is causative or accidental.

Given that both autophagy activation and inhibition have been found to increase the effectiveness of anti-cancer medications, the role of autophagy in cancer therapy requires further clarification. Therefore, the key question is whether we should strive to increase or decrease autophagy while treating cancer. Additionally, exploring the regulatory mechanisms of autophagy needs to consider that degradative autophagy blocking is followed by induction and regulation of secretory autophagy. These investigations may help to coordinate the effect of autophagy modulators better. To maximize patient benefit and enhance cancer treatment, precision-targeted medications should be further studied. Of note, the currently utilized autophagy inhibitors are not especially effective at enhancing anti-tumor immunity. Thus, research is needed to clarify the appropriate dose, target, time, marker, and tumor type to maximize their anti-tumor activity and sensitize immunotherapy.

## Author contributions

ZJ, XS contributed to the data collection and writing the manuscript. YW, CZ made the figures and tables. SZ, and HY designed and guided this study and edited the manuscript. All authors listed made a substantial, direct, and intellectual contribution to the work and approved it for publication. All authors contributed to the article and approved the submitted version.

## Funding

This study was supported by National Natural Science Foundation of China (NSFC 81872458), and Natural Science Foundation of Zhejiang Province (LY19H160017).

## Acknowledgments

We appreciate the researchers and study participants for their contributions.

## Conflict of interest

The authors declare that the research was conducted in the absence of any commercial or financial relationships that could be construed as a potential conflict of interest.

## Publisher’s note

All claims expressed in this article are solely those of the authors and do not necessarily represent those of their affiliated organizations, or those of the publisher, the editors and the reviewers. Any product that may be evaluated in this article, or claim that may be made by its manufacturer, is not guaranteed or endorsed by the publisher.

## Glossary

**Table:**

3-MA	3-methyladenine
AAK1	Adaptor-associated kinase 1
AMPK	AMP-activated protein kinase
APC	antigen-presenting cell
ATG	autophagy-related gene
ATP	adenosine triphosphate
BNIP3	adenovirus E1B 19 kDa-interacting protein 3
BNIP3L	adenovirus E1B 19 kDa-interacting protein 3-like
CAF	cancer-associated fibroblasts
CAR	chimeric antigen recepter
CAER	chemotherapeutic medicines plus the autophagy enhancer rapamycin
CDDP	cisplatin
CQ	chloroquine
CTL	cytotoxic T lymphocyte
CTLA4	Cytotoxic T lymphocyte–associated protein 4
DAMP	damage-associated molecular pattern
DC	dendritic cells
DRiPs	Defective ribosome products
DRibbles	defective ribosomal products in blebs
EC	endothelial cell
ECM	extracellular matrix
ER	Endoplasmic
FDA	The US Food and Drug Administration
G-CSF	granulocyte colony-stimulating factor
GM-CSF	granulocyte-macrophage colony-stimulating factor
HCQ	hydroxychloroquine
HMGB1	high-mobility group box 1
HIF-1	hypoxia-inducible factor-1
ICIs	immune checkpoint inhibitors
ICD	immunogenic cell death
IFN	interferon
IL	interleukin
MAP	microtubule-associated
MDSC	myeloid-derived suppressor cells
MHC	major histocompatibility complex
mTOR	mammalian target of rapamycin
MVB	Multivesicular body
NF-κβ	nuclear factor κβ
NK	natural killer
NSCLC	non-small cell lung cancer
PD-1	programmed cell death 1
PD-L1	programmed cell death ligand 1
Peg3	paternally expressed gene 3
PEM	Pemetrexed
PI3K	Phosphoinositide 3-kinase
PtdIns3K	class III phosphatidylinositol 3-kinase complex
SLiPs	short-lived proteins
SQSTM1	sequestosome-1
TAM	tumor-associated macrophages
TGF	transforming growth factor
TME	tumor microenvironment
TNFα	Tumor necrosis factor alpha
Treg	regulatory T cells
TRAP	Tumor cell-released autophagosomes
ULK1	unc-51-like autophagy-activated kinase 1
VEGF(R)	vascular endothelial growth factor (receptor)
